# 

**Published:** 1900-05-15

**Authors:** 


### ANNOUNCEMENTS.

THE Pennsylvania State Dental Society has changed its date of meeting to July 5th, 6th and 7th, at Reading, Pa.

THE Section on Etiology and Physiology, of the National Dental Association, desires that those proposing to read papers before this Section should, at the earliest date, notify the Secretary of the Section,

DR. L. E. CUSTER,
Dayton, Ohio.

AT the last meeting of the National Dental Association it was decided by Section III. to make the work of this section a feature of the meeting. To this end it has been arranged to hold the meetings of this section at such times as will not interfere with the general sessions. *All papers* upon the subjects embraced in this section will be read in these meetings excepting two or three, which, from their general interest, have been selected by the committee for presentation to the general body.

A suitable room will be provided and the program for each meeting duly announced. Some good papers are promised.

It was further decided to hold clinics. These will comprise operations upon patients, and demonstrations upon casts, models, etc. It is desired that everyone who has anything new, original and helpful will bring or send his appliances, models and illustrations. While new appliances may be shown subject to the provisions of the Constitution, nothing can be offered for sale. Suitable provision will be made for the carrying out of these clinics.

Let everyone interested in this section who has anything to offer communicate at once with

THOS. E. WEEKS, *Chairman*,
Dayton Bldg., Minneapolis, Minn.,
JNO. I. HART, *Secretary*,
118 West 55th Street, N. Y.,
or THOS. P. HINMAN,
*Chairman Clinic Com.*, Atlanta, Ga.

